# The use of negative pressure wound therapy for fracture-related infections following internal osteosynthesis of the extremity: A systematic review

**DOI:** 10.1016/j.jcot.2021.101710

**Published:** 2021-11-17

**Authors:** Niels Martin Jensen, Signe Steenstrup, Christen Ravn, Hagen Schmal, Bjarke Viberg

**Affiliations:** a Department of Orthopaedic Surgery and Traumatology, Kolding Hospital Part of Lillebaelt Hospital, Sygehusvej 22, 6000, Kolding, Denmark; b Department of Orthopaedic Surgery and Traumatology, Odense University Hospital, J. B. Winsloewsvej 4, 5000, Odense C, Denmark; c Department of Orthopaedic Surgery and Traumatology, Freiburg University Hospital, Hugstetter Straße 55, 79106, Freiburg, Germany

**Keywords:** NPWT, negative pressure wound therapy, FRI, fracture-related infection, NPWT, FRI, Dressing, Osteosynthesis

## Abstract

This study aimed to systematically review the current literature on studies using negative pressure wound therapy (NPWT) or dressings following fracture-related infection (FRI) in internal osteosynthesis of the extremity. Articles were analyzed on fracture and wound healing and included when comparing or describing the use of either NPWT or dressings in FRI. We conducted a systematic literature search in four electronic databases: Embase, Medline, the Cochrane Library, and Scopus. The studies were screened by two authors using Covidence.org and evaluated for risk of bias. A total of 8576 records were identified. No articles compared NPWT to dressings. Seven case reports and three case series included a total of 115 patients treated for FRI. Fracture healing was achieved in 21 out of 67 patients treated with NPWT (4 amputations and 46 not described) and all 48 patients in the dressing group (4 patients needed additional sequestrectomy procedures). Five studies did not describe fracture healing. In 57 out of 67 patients treated with NPWT, the wounds were described as healed, closed, or requiring soft tissue reconstruction (4 amputations and six lacking description). The dressing group had complete wound coverage in 18 patients and partial coverage in 30 patients. Studies were generally at high risk of bias because of insufficient descriptions of both patient demographics and outcomes. No studies compared NPWT to dressings, and the existing literature is at high risk of bias. The included studies were of low-level evidence. NPWT can be neither recommended nor advised against to cover infected osteosynthesis.

## 1 Introduction

Fracture-related infection (FRI) in internal osteosynthesis of the extremity is a limb-threatening complication of fracture treatment.^1^ Treatment options for FRI are versatile, including antibiotic suppression while the bone heals, debridement and antibiotic treatment, debridement and reosteosynthesis, or implant removal with antibiotic suppression. Removing the implant before healing can cause an unstable fracture, increase the risk of nonunion, and accelerate the ongoing infection.^1^ This raises the question of the possibilities in FRI when retaining the implant is desired.

Fracture healing requires vital tissue, but the most important step in infection control is debridement of the soft tissue. Thorough debridement potentially causes soft tissue defects and thus exposes the fracture and osteosynthesis material. This causes delayed wound healing and increased risks of contamination and reinfection, as well as accelerated development of bone and soft tissue necrosis.^2^ Applying a physical barrier between the osteosynthesis material and the surrounding environment is crucial to prevent bacteria from entering. Reconstructive surgery with flap coverage is a well-known method to cover wounds,^3^ but not all patients are candidates for this treatment. Reconstructive surgery is also not available in all hospitals. Another well-known and simpler barrier is applying a dressing to covering the defect.^4^ Numerous treatment options for dressings have been suggested, but none with any superior results.^4^ Another treatment option gaining increasing interest is negative pressure wound therapy (NPWT).^5^ NPWT ensures that the wound is closed with an airtight dressing from which excessive fluid is actively drained.^5^ It has not been shown to improve the healing of traumatic open wounds or fractures but has been demonstrated to yield faster wound healing in the treatment of chronic foot ulcers.^2^^,^^6^ NPWT has resulted in fewer reconstruction flaps^7^ and potentially fewer amputations in open tibia–fibula fractures. This study aimed to systematically evaluate the current literature on studies using NPWT or dressings in the treatment of FRI in internal osteosynthesis of the extremity. The primary objective was to investigate the effect on fracture healing of NPWT in comparison to dressings following FRI in internal osteosynthesis of the extremity. The secondary objective was to investigate the fracture healing time, wound healing, implant removal, rate of amputation, time in hospital, quality of life, rate of reconstructive surgery, and cost-effectiveness.

## 2 Materials and methods

### 2.1 Protocol and registration

This systematic review is reported according to the Preferred Reporting Items for Systematic reviews and Meta-Analyses (PRISMA) statements.^8^ The study protocol was registered in the International Prospective Register of Systematic Reviews (PROSPERO) before data extraction (registration number CRD42020199605).^9^

### 2.2 Eligibility criteria

The PICO model was used to create the research question: (P)articipants were patients with fracture related infections following internal osteosynthesis of fracture of the extremity; (I)ntervention was wound cover by NPWT; (C)omparator was all types of dressings; (O)utcome was fracture healing. Dressings were defined as all dressings other than NPWT, such as plain gauze, bandages and medicated bandages. The definition of fracture related infections is based on the algorithm described by Metsemakers et al. from 2018 for fractures treated with internal osteosynthesis.^10^ The secondary outcomes were fracture healing time, wound healing, implant removal, rate of amputation, quality of life, rate of reconstructive surgery, and cost-effectiveness. The inclusion criteria were published studies and patients over 15 years of age with a fracture related infection following internal osteosynthesis of a fracture of the extremity treated with NPWT, which include all infections needing coverage of dressings or NPWT with or without surgical debridement. The exclusion criteria were animal and cadaver studies; fractures treated with prostheses; face, head, neck, spine, and thoracic fractures; tumor or cancer surgery; external fixation; arthrodesis; and languages other than English, German, or Danish.

### 2.3 Information sources

We conducted a systematic literature search in four electronic databases: Embase, Medline, the Cochrane Library, and Scopus. The European Bone and Joint Infection Society^11^ and European Wound Management Association homepages were also searched for studies, but no further studies were included.^12^

### 2.4 Search

The search strategy was developed in collaboration with a scientific research librarian from the University of Southern Denmark. The search was made on both MeSH terms and free-text words in three blocs with synonyms for NPWT, dressing, osteosynthesis, and infection. The Boolean operator “AND” was used to combine the three blocs: “NPWT AND osteosynthesis AND infection” or “dressing AND osteosynthesis AND infection.” The Boolean operator “OR” was used between synonyms in each bloc. See Appendices A and B for the complete search string.

The search limitations were publications until 2021 in Scopus, April 2020 in the Cochrane Library, and April 17th, 2020, in Medline and Embase. The last search was performed on February 5th, 2021.

### 2.5 Study selection

The initial plan was to evaluate studies comparing NPWT to dressings following FRI in internal osteosynthesis of the extremity to perform a meta-analysis. In the initial literature screening, no such studies were found. We therefore decided to change the direction of the study towards any studies describing the use of either NPWT or dressings following FRI in internal osteosynthesis of the extremity.

The records were imported to EndNote to search for duplicates, then imported to Covidence (Veritas Health Innovation, Melbourne, Australia; available at www.covidence.org) for screening. The records were screened based on title and abstract, independently and blinded by the two main authors. The included studies were then full-text screened by the same two authors. Any disagreements were resolved by consultation with the senior author.

### 2.6 Data collection process

The data were extracted into an Excel sheet (Microsoft® Excel for Mac, Office 365 version 16.44) by one author and verified by another author. Any disagreements were resolved by a senior author.

For additional data, five authors were contacted,^13^, ^14^, ^15^, ^16^ and one^17^ responded with an anonymized datasheet. The data extracted resulted in an additional 51 patients for inclusion.

### 2.7 Data items

The variables registered for each study were title, author, year, patients in study, patients for inclusion, fracture type, age of patients (years), fracture healing, osteosynthesis, intervention, NPWT vacuum, vacuum flow, duration of NPWT, period of changes in NPWT, wound outcome, time to wound healing, time to reconstructive surgery, time to fracture healing, amputations, bacteria, type of antibiotics, days with antibiotics, wound and infection description, implant management, health-related quality of life, cost-effectiveness, and definition of FRI.

### 2.8 Risk of bias in individual studies

The risk of bias was assessed using the Critical Appraisal Checklist for Case Series and Case Reports from The Joanna Briggs Institute.^18^ Outcomes were presented as Yes, Unclear, or No in accordance with the checklist. Data were plotted into an Excel sheet (Microsoft® Excel for Mac, Office 365 version 16.44) by one author and verified by another author.

### 2.9 Statistics and synthesis of results

No summary measures or meta-analysis could be performed because the data presented great heterogeneity.

## 3 Results

### 3.1 Study selection

A total of 8576 records were identified; after removal of duplicates, 6543 studies were screened. No articles compared NPWT to dressings following FRI in internal osteosynthesis of the extremity.

**Fig. 1:**
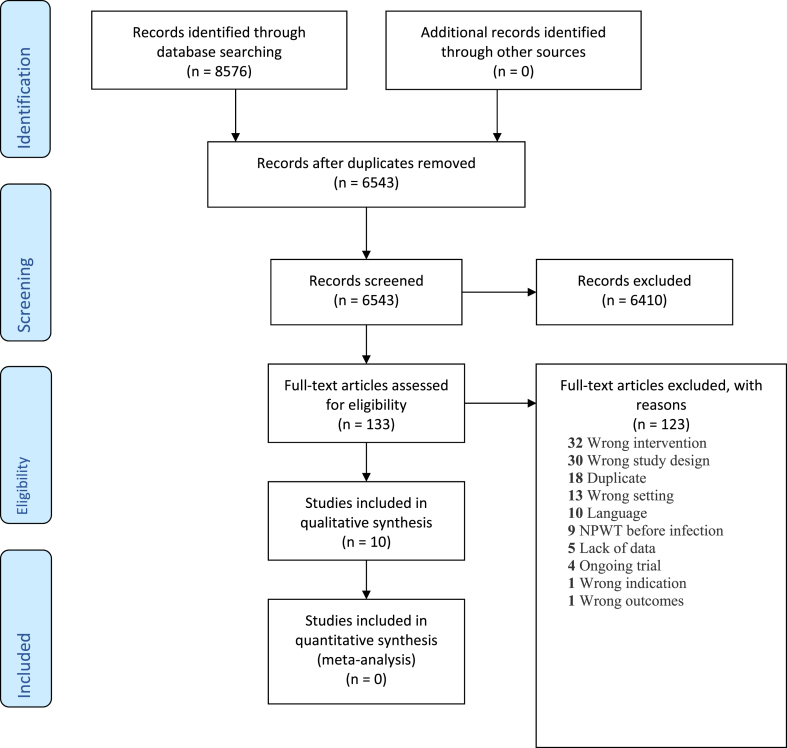
PRISMA flow diagram.

Ten studies included either NPWT or dressings following FRI in internal osteosynthesis of the extremity and could therefore be included in the review (Fig. 1).

### 3.2 Included studies

The studies comprised seven case reports^19^, ^20^, ^21^, ^22^, ^23^, ^24^, ^25^ and three case series.^17^^,^^26^^,^^27^ Patient ages ranged from 12 to 83 years (Table 1). One study group had an age range of 12–61 years, and only three patients met the inclusion criteria, but the ages of these patients were not described further. One study included fractures of the upper extremity,^17^ and eight focused on lower leg fractures.^19^, ^20^, ^21^, ^22^, ^23^, ^24^, ^25^, ^26^ One study did not specify the anatomical region but included solely shaft fractures.^27^ The ten studies had a total of 201 patients. However, 86 patients in the ten studies did not meet the inclusion criteria (e.g., external fixation, no fracture, no infection, no osteosynthesis, prosthesis, and spine fracture), so only data on 115 patients could be analyzed (Table 1), 67 patients treated with NPWT and 48 patients treated with dressings.

**Table 1:** Study demographics.

Author	Year	Study design	Age (years)^b^	No. in study	No. for inclusion	Patient exclusion reasons
Anagnostakos et al.^19^	2006	Case report	58	6	1	Prosthesis, spine fracture, no fracture
Grecu et al.^20^	2017	Case report	59	1	1	None excluded
Izadpanah et al.^17^	2017	Case series	20–83	106	51	External fixation, no fracture, spine fracture
Kollrack et al.^21^	2012	Case report	58–67	7	6	External fixation
Marinovic et al.^22^	2014	Case report	35	1	1	None excluded
Rawicki et al.^26^	2015	Case series	12-61^a^	17	3	No infection
Roth et al.^27^	1997	Case series	Not described	48	48	None excluded
Sharp et al.^23^	2013	Case report	33, 83	10	2	No osteosynthesis, Amputation before NPWT, no fracture, prosthesis, external fixation
Wijewardena et al.^24^	2011	Case report	24	4	1	NPWT after reconstructive surgery, no osteosynthesis
Windhofer et al.^25^	2009	Case report	46	1	1	None excluded

a The study group had an age range of 12–61 years. Only three patients met the inclusion criteria, but the age of these patients were not described.

b Total age and range.

### 3.3 Study intervention

NPWT was described differently depending on the device available. Five studies described NPWT on wound defects, one study on wound breakdown, one on fistulas, one on unspecified deep wound infection, and one on swelling, redness, and pain (Table 2).

**Table 2:** Wound treatment.

Author	Wound outcome	Time for wound healing^a^	Wound and infection description	Time for infection after osteosynthesis^a^
Anagnostakos et al.^19^	Secondary closure	Not described	Infected wound defect with visible implant	3 weeks
Grecu et al.^20^	Sural fasciocutaneous flap 28 days after NPWT	31 days	Infected wound defect with visible implant	4 days
Izadpanah et al.^17^	Granulation in 1, secondary wound closure in 24, plastic reconstruction in 17, amputation in 4, unknown in 5	Not described	Persistent infection or insufficient soft tissue coverage following open reduction and internal fixation, local infection signs, leukocytes and CRP	2–341 days
Kollrack et al.^21^	Mesh graft	16.3 days	Sepsis with infected osteosynthesis	38–51 days
Marinovic et al.^22^	Wound healed 10 days after NPWT	10 days	Skin defect, visible implant, secretion, fever, CRP, leukocytes, SR, pain	2 months
Rawicki et al.^26^	Wound healed within 2.5 months	Not described	Deep wound infection, not described if implant was visible	Not described
Roth et al.^27^	Implant cover in 18 patients, and partial implant cover in 30 patients	Not described	Fever, pain, swelling, redness	From 3 days to several months
Sharp et al.^23^	NPWT discontinued in one patient and wound healed in the other patient	Not described and 2 months	Wound breakdown, not described if implant was visible	1 month
Wijewardena et al.^24^	Skin graft	108 days	Infected wound defect with visible implant	Not described
Windhofer et al.^25^	Tensor fascia lata flap	2 months	Purulence throughout fistula, local and systemic infections signs	29 days

a Total days/months and range.

Vacuum pressure ranged from 80 mmHg to 150 mmHg, with continuous, intermittent, and combination flow modes. One study used NPWT for periods from zero to more than 50 days, whereas the other studies used NPWT for four to 108 days. The NPWT was changed somewhere between every two to seven days (Table 3).

**Table 3:** Wound intervention.

Author	Intervention described	NPWT vacuum	Vacuum flow	Period with NPWT	Period between exchange of NPWT
Anagnostakos et al.^19^	Debridement, NPWT, antibiotics	125–150 mmHg	Continuous	30–50 days	3–5 days
Grecu et al.^20^	Debridement, Platelet Rich Plasma, NPWT, antibiotics	140 mmHg	Continuous, then intermittent	28 days	48 h
Izadpanah et al.^17^	Debridement, NPWT, antibiotics	Not described	Not described	From 0 to more than 50 days	4–5 days
Kollrack et al.^21^	Debridement, NPWT	125 mmHg	Continuous, then intermittent	53–57 days	3–4 days
Marinovic et al.^22^	Debridement, NPWT, antibiotics	125 mmHg	Continuous, then intermittent	10 days	5 days
Rawicki et al.^26^	Debridement, NPWT, antibiotics	Not described	Not described	2.5 months	Not described
Roth et al.^27^	Debridement, antiseptic dressing	–	–	–	–
Sharp et al.^23^	2 NPWT, 1 antibiotics	80 mmHg	Not described	1 and 3 weeks	7 days
Wijewardena et al.^24^	NPWT, activated protein C	125 mmHg	Intermittent, 5 min on, 2 min off	3 months and 18 days	Not described
Windhofer et al.^25^	Debridement, NPWT, antibiotics	Not described	Not described	4 days	Not described

Fifty-seven out of 67 patients were treated with debridement, NPWT, and antibiotics. Six patients were treated solely with debridement and NPWT, and one patient was treated with NPWT and antibiotics. One patient was treated with debridement, platelet-rich plasma, NPWT, and antibiotics. One patient was treated with NPWT alone, and one patient was treated with NPWT and activated protein C (Table 3). Of the 67 patients treated with NPWT, osteosynthesis was retained in 25, exchanged in 14, and removed in 27; information was missing for one (Table 4).

**Table 4:** Fracture and osteosynthesis management.

Author	Fracture type	Osteosynthesis	Osteosynthesis management	Fracture healing	Fracture healing time
Anagnostakos et al.^19^	Fibula	Plate, screws, unknown locking	Retained	Not described	Not described
Grecu et al.^20^	Tibial malleolus	Plate, screws, unknown locking	Retained	Not described	Not described
Izadpanah et al.^17^	Clavicle, humerus, forearm, femur, patella, tibia, calcaneus	Plate, nail, wires	23 removed, 14 exchanged, 14 retained	13 healed, 4 amputated, 34 not described	Not described
Kollrack et al.^21^	Ankle	Plate, screws, unknown locking	2 removed, 4 retained	Yes, all fractures healed	8 weeks
Marinovic et al.^22^	Tibia	Plate, screws, locking	Retained	Yes	12 months
Rawicki et al.^26^	Intraarticular calcaneal	Internal fixation	2 removed, 1 retained	Not described	Not described
Roth et al.^27^	Shaft fracture	Plate, screws, unknown locking	48 removed	44 spontaneous, 4 sequestrotomy	Not described
Sharp et al.^23^	Tibia, Tibial plafond	Internal fixation	1 not described, 1 retained	Not described	Not described
Wijewardena et al.^24^	Metatarsal	Plate, screws, unknown locking	Retained	Not described	Not described
Windhofer et al.^25^	Infracondylar tibia	Plate, screws, locking	Retained	Yes	170 days

The study by Roth et al. was the only study using dressings.^27^ In all 48 patients, the wounds were debrided and covered with gauze strips moistened with antiseptic 0.1% hexamethylene biguanide solution (Lavasept®).^27^ Eight out of 48 patients were treated with systemic antibiotics because of fever or signs of infection. Wound and infection descriptions included fever, pain, swelling, and redness.

### 3.4 Fracture healing

Fracture healing was achieved in 21 out of 67 patients in four studies with NPWT, four patients were amputated,^17^^,^^21^^,^^22^^,^^25^ and 42 patients had no description of fracture healing.^17^ Five studies did not describe fracture healing.^19^^,^^20^^,^^23^^,^^24^^,^^26^ In the dressing group, fracture healing was described as spontaneous in 44 cases, and four required additional sequestrectomy procedures.^27^

The time to fracture healing was only described in eight out of 67 patients treated with NPWT.^21^^,^^22^^,^^25^ Fractures healing time was described after 170 days for one patient in one study,^25^ 8 weeks in six patients in another study,^21^ after 12 months for one patient in a third study.^22^ However, the time for fracture healing was not described in the remaining six studies. The time to fracture healing was not described for patients with dressing treatment [26] (Table 4).

The time from osteosynthesis to infection was 2–341 days and was described in eight studies.^17^^,^^19^, ^20^, ^21^, ^22^, ^23^^,^^25^^,^^27^ We cannot confirm whether all fractures were unhealed before infection since this was not described for all patients. For NPWT, implant management was not described for one patient, retained in 25 patients, removed in 75 patients, and exchanged in 14 patients.^17^^,^^19^, ^20^, ^21^, ^22^, ^23^, ^24^, ^25^, ^26^ In the dressing study, all 48 patients had their implants removed.^27^

### 3.5 Wound healing

Wound breakdown and protruding metal were the most frequently used descriptions of wound healing failure. In 57 out of 67 patients treated with NPWT, the wounds were described as healed, closed, or requiring additional soft tissue reconstruction (Table 2). Of the remaining ten patients, four were amputated, and wound healing was not described for six. Seventeen out of 67 patients treated with NPWT had additional reconstructive surgery. In 40 out of 50 patients (80%) treated with NPWT, the wounds were described as healed or covered without reconstructive surgery. One patient discontinued NPWT after 7 days due to a lack of regression of the wound bed. In the study with antiseptic dressings by Roth et al. complete wound coverage was achieved in 18 patients and partial coverage in 30 patients; thus, 18 out of 48 patients (38%) had complete wound coverage. The degree of coverage was evaluated at the time of implant removal and not described further.^27^

Five NPWT studies described the time to wound healing or closure ranging from ten to 108 days.^20^, ^21^, ^22^, ^23^, ^24^ Two NPWT studies described the time to reconstructive surgery after 14 and 21 days.^20^^,^^25^ Two patients treated with NPWT were described as diabetic.^26^ In the dressing study, only complete or partial wound coverage of osteosynthesis was described at removal. Two out of 48 patients had persistent fistulas after removal of the osteosynthesis at three and 11 years of follow-up, respectively.

### 3.6 Secondary outcomes

*Staphylococcus aureus* was the most frequently occurring bacterial strain, although not all studies described the strain (Table 5). The type of antibiotics used varied, and the treatment period ranged from 10 days to 7 weeks. Not all studies described the type of antibiotics or route of administration.

**Table 5:** Bacterial strains and antibiotics.

Author	Bacteria	Type of antibiotics	Period of antibiotics
Anagnostakos et al.^19^	*S. marcescens*, *S. aureus*	Flucloxacillin, clindamycin, levofloxacin, unknown administration path	Not described
Grecu et al.^20^	Not described	Ceftriaxone 2 g/day	10 days
Izadpanah et al.^17^	MRSA, pseudomonas aeroguinosa, staphylococcus aureus, Enterococcus faecium, Bacillus species, staphylococcus epidermidis, streptococcus equisimilis, enterobacter cloacae, stentrophomonas maltoplillae, acinetobacter baumannii, VRE, Peptostreptococcus Species, Streptococcus agalagticae	cephalosporin or bacteria-specific	Not described
Kollrack et al.^21^	*S. aureus*, Enterococcus	Not described	Not described
Marinovic et al.^22^	Staphylococcus spp.	Azithromycin	Not described
Rawicki et al.^26^	*Staphylococcus aureus* or *Serratia marcescens*	Zosyn and Rifampin or Ceftriaxone, via peripherally inserted central catheter	Not described
Roth et al.^27^	Not described	8 patients treated with systemic antibiotics if fever or generalized infection signs	Not described
Sharp et al.^23^	Not described	Intra venous for one patient	Up to 6 weeks
Wijewardena et al.^24^	Not described	None	None
Windhofer et al.^25^	*Staphylococcus aureus* and epidermidis	1. stage (Clindamycin and vancomycin), 2. stage (levofloxacin and doxycyclin)	Stage 1 for 4 weeks and stage 2 for 3 weeks

No studies described the health-related quality of life, cost-effectiveness, or definition of FRI.

### 3.7 Risk of bias

The included studies were critically assessed using the Critical Appraisal Checklist for Case Series and Case Reports from The Joanna Briggs Institute.^18^ The majority had a high risk of bias, as presented in Table 6, Table 7. No case reports described the demographic characteristics of the patients, and only two case reports included a sufficient description of the diagnostic tests used, methods, and results. No case series described their outcomes in a standard, valid, and reliable way.

**Table 6:** Risk of bias case reports.

Author	Year	Question 1	Question 2	Question 3	Question 4	Question 5	Question 6	Question 7	Question 8
Anagnostakos et al.^19^	2006	Unclear	No	Unclear	Unclear	Unclear	Unclear	Unclear	No
Grecu et al.^20^	2017	Unclear	Yes	Unclear	Yes	Yes	Unclear	Yes	No
Kollrack et al.^21^	2012	Unclear	Unclear	Yes	Unclear	Unclear	Yes	Yes	Yes
Marinovic et al.^22^	2014	Unclear	Yes	Yes	Unclear	Yes	Yes	No	Unclear
Sharp et al.^23^	2013	Unclear	Unclear	Unclear	No	Unclear	Unclear	Unclear	Yes
Wijewardena et al.^24^	2011	Unclear	Unclear	Unclear	No	Unclear	Unclear	Yes	Unclear
Windhofer et al.^25^	2009	Unclear	Yes	Unclear	Unclear	Unclear	Unclear	No	No

Data plotted as Yes (green) shows low risk of bias, Unclear (yellow) as moderate risk of bias, and No (red) as high risk of bias. Question 1–8 is described in Appendix C.

**Table 7:** Risk of bias case series.

Author	Year	Question 1	Question 2	Question 3	Question 4	Question 5	Question 6	Question 7	Question 8	Question 9	Question 10
Izadpanah et al.^17^	2017	Yes	No	No	Yes	Yes	Unclear	Unclear	Unclear	No	Yes
Rawicki et al.^26^	2015	Unclear	No	No	Yes	Yes	No	No	No	No	No
Roth et al.^27^	1997	Unclear	No	No	Unclear	Unclear	No	No	Unclear	No	No

Data plotted as Yes (green) shows low risk of bias, Unclear (yellow) as moderate risk of bias, and No (red) as high risk of bias. Question 1–10 is described in Appendix D.

## 4 Discussion

This is the first systematic review on the use of NPWT and dressings following FRI in internal osteosynthesis of the extremity. We did not find any studies comparing the use of NPWT with dressings following FRI. We found ten studies with a total of 115 patients with FRI treated with either NPWT or dressings. Fracture healing was described in less than one third of the patients, and the time to fracture healing was only described in seven out of 115 patients. The data were therefore too small to show an effect of NPWT versus dressings. Debridement was used in eight out of ten studies, which shows that this is a common step in the treatment of FRI.

Fracture healing is one of the main issues related to FRI and therefore the primary outcome in this review. The fracture location and type of osteosynthesis varied substantially in the studies, which could influence both fracture and wound healing. Osteosynthesis with plate and screws was described in eight out of ten studies, which could influence fracture healing since periosteal stripping might occur and thereby disturb the blood supply.^28^ Bones surrounded with vital tissue such as muscles have a better blood supply and thereby better fracture healing.^28^ Large wound defects at the fracture site may increase the risk of infection and compromised fracture healing, and therefore wound healing was our secondary outcome.

A limitation of this review is that the majority of the studies are case reports and therefore present low-level evidence. Case reports are mostly published with positive data or data that presents an effect of treatment and seldom negative data or no effect of treatment. This increases the risk of publication bias. The internal validity of this review is low because of the lack of transparency in the included studies, which is reflected by the poorly described patient demographics and confounders in the case reports. Smoking and diabetes are known risk factors for fracture healing^29^^,^^30^ but were only described in one study for two patients.

Most studies used a vacuum force of 125 mmHg, but this ranged from 80 to 150 mmHg and was not discussed in any studies. Further, some studies used both continuous and intermittent vacuum. Studies have described the risk of patient discomfort with the use of intermittent vacuum.^31^^,^^32^ These studies did not show increased wound contraction with vacuum higher than 75 mmHg, but more fluid drainage was noticed at 125 mmHg.^31^^,^^32^ Therefore, the vacuum force should be adjusted in accordance with the desired result. Animal studies have shown both no difference and more granulation tissue when comparing continuous versus noncontinuous NPWT.^31^^,^^33^^,^^34^ Consensus on this is therefore difficult. Additionally, studies using healthy young swine with well-defined clean wounds,^31^^,^^33^ which would normally heal on their own, are difficult to extrapolate to humans with infected irregular wounds with unhealed fractures and metal implants. Therefore, comparable studies are needed with different vacuum forces and continuous versus noncontinuous vacuum on infected human wounds.

The European Wound Management Association has published a compendium on the use of NPWT in visible osteosynthesis postoperatively.^35^ The recommendations include that NPWT can be used when covering the exposed metalwork is otherwise not possible.^35^ In addition, they state that NPWT should be used as a last attempt to prevent amputation.^35^ These recommendations rely on two studies: one on NPWT with exposed bone^36^ and one on an experimental model with porcine wounds.^31^ Thus, they do not rely on research on fractures with exposed metalwork. This amplifies the need for further studies on the use of NPWT following FRI with exposed internal osteosynthesis.

None of the included studies clearly defined FRI following internal osteosynthesis. Overall, a consensus is lacking on the definition, which has been described previously.^10^ A clear definition would enable easier comparison between studies. In addition, a definition of FRI would help surgeons decide whether an osteosynthesis is infected and when to surgically intervene, similar to the procedure for infected arthroplasties.^37^ In 2019, Govaert et al. published an FRI consensus definition that offered a guideline for surgeons to improve the comparison and quality of published literature.^38^

Although we included as many studies as possible given the language skills of our authors, many studies in Chinese were rejected, contributing to selection bias. Therefore, studies that compare NPWT to dressings may exist in other languages than those included in our study.

The use of the PRISMA statement makes this study systematic and transparent. All literature and data extraction was systematically and critically reviewed and evaluated for risk of bias by two authors, which strengthens the study. To find additional grey literature, we searched The European Bone and Joint Infection Society homepage^11^ and The European Wound Management Association homepage.^12^

We found few studies overall, with few participants, generally inferior quality, no identical definition on the indication for surgery, and no comparison of NPWT to dressings. This systematic review clarifies the need for studies to answer these questions. To raise the level of evidence, multicenter randomized clinical trials on a larger scale are desired with a clear definition and indication for surgery comparing NPWT with dressings to treat FRI in internal osteosynthesis of the extremity. This could include more patients and thereby stratify these, contributing to more transparency in which patients might benefit from these different treatment options.

Regarding the external validity and real-life applicability of this review, it is difficult to extrapolateing the findings into general recommendations is difficult because of the few included studies with low level of evidence and high risk of bias.

## 5 Conclusions

This study aimed to systematically evaluate the current literature on studies comparing NPWT with dressings following fracture-related infection in internal osteosynthesis of the extremity. No articles compared the two treatment methods. Few studies were found, with few patients and a low level of evidence. Fracture healing was rarely described, and wound healing was described more frequently but not enough to make an adequate comparison. No scientific evidence exists to recommend or advise against the use of NPWT to cover infected internal osteosynthesis materials, based on this systematic review.

## CRediT authorship contribution statement

**Niels Martin Jensen:** Conceptualization, Data curation, Formal analysis, Investigation, Methodology, Project administration, Validation, Visualization, Writing – original draft, Writing – review & editing. **Signe Steenstrup:** Conceptualization, Formal analysis, Investigation, Methodology, Validation, Writing – review & editing. **Christen Ravn:** Conceptualization, Methodology, Validation, Supervision, Writing – review & editing. **Hagen Schmal:** Conceptualization, Methodology, Validation, Supervision, Writing – review & editing. **Bjarke Viberg:** Conceptualization, Methodology, Validation, Supervision, Writing – review & editing.

## Declaration of competing interest

None.
