# Supplementary material for: The use of negative pressure wound therapy for fracture-related infections following internal osteosynthesis of the extremity: A systematic review
Source: J Clin Orthop Trauma. 2021 Nov 17;24:101710. doi: 10.1016/j.jcot.2021.101710 (PMC8627993; doi:10.1016/j.jcot.2021.101710)
Supplement: Supplementary file 1 [file mmc1.docx]

# Appendix

# Appendix A – Search synonyms

## Cochrane Library

**NPWT**

MeSH descriptor: [Negative-Pressure Wound Therapy] explode all trees

MeSH descriptor: [Suction] explode all trees
MeSH descriptor: [Vacuum] explode all trees

((npwt) OR (negative*pressure* NEXT wound NEXT therap*) OR (negative*pressure* NEXT dressing*) OR (topical* NEXT negative*pressure* NEXT therap*) OR (vac) OR (vacuum*) OR (suction*)):ti,ab,kw

**Dressing**

MeSH descriptor: [Bandages] explode all trees

MeSH descriptor: [Surgical Sponges] explode all trees

((dressing*) OR (bandage*) OR (jelonet*) OR (gauze*) OR

(surgical NEXT sponge*)):ti,ab,kw

**Osteosynthesis**

MeSH descriptor: [Fracture Fixation, Internal] explode all trees

MeSH descriptor: [Fracture Fixation] explode all trees
MeSH descriptor: [Fracture Fixation, Intramedullary] explode all trees

MeSH descriptor: [Internal Fixators] explode all trees
MeSH descriptor: [Bone Plates] explode all trees
MeSH descriptor: [Bone Screws] explode all trees
MeSH descriptor: [Bone Nails] explode all trees

MeSH descriptor: [Bone Wires] explode all trees

((osteosynthes*) OR (fracture NEXT fixat*) OR (skelet* NEXT fixat*) OR (fracture* NEXT reduction*) OR (intramedullar* NEXT nail*) OR (internal NEXT fixat*) OR (bone* NEXT plat*) OR (bone* NEXT screw*) OR (bone* NEXT nail*) OR (bone* NEXT pin*) OR (fracture* NEXT plat*) OR (fracture* NEXT screw*) OR (fracture* NEXT nail*) OR (fracture* NEXT pin*) OR (skelet* NEXT plat*) OR (skelet* NEXT screw*) OR (skelet* NEXT nail*) OR (skelet* NEXT pin*) OR (bone* NEXT wire*) OR (k*wire*) OR (kirschner NEXT wire*) OR (cerclage) OR (tension NEXT band*) OR (rod*) OR (stabil*)):ti,ab,kw

**Infection**

MeSH descriptor: [Bone Diseases, Infectious] explode all trees

MeSH descriptor: [Sepsis] explode all trees
MeSH descriptor: [Soft Tissue Infections] explode all trees

MeSH descriptor: [Suppuration] explode all trees
MeSH descriptor: [Abscess] explode all trees
MeSH descriptor: [Coinfection] explode all trees
MeSH descriptor: [Wound Infection] explode all trees
MeSH descriptor: [Surgical Wound Infection] explode all trees

MeSH descriptor: [Equipment Contamination] explode all trees

MeSH descriptor: [Infection] explode all trees

MeSH descriptor: [Osteomyelitis] explode all trees

MeSH descriptor: [Suppuration] explode all trees

((infection*) OR (infect* NEXT bone*) OR (fracture* NEXT infect*) OR (sepsis) OR (blood NEXT poisoning*) OR (soft NEXT tissue* NEXT infection*) OR (suppuration*) OR (pus*) OR (abscess*) OR (co*infection*) OR (coinfect*) OR (equipment* NEXT contamination*) OR (osteomyelit*)):ti,ab,kw

## Medline

**NPWT**

exp Negative-Pressure Wound Therapy/
exp Vacuum/
exp Suction/

(npwt or negative*pressure* wound therap* or negative*pressure* dressing* or topical* negative*pressure* therap* or vac or vacuum* or suction*).mp. [mp=title, abstract, original title, name of substance word, subject heading word, floating sub- heading word, keyword heading word, organism supplementary concept word, protocol supplementary concept word, rare disease supplementary concept word, unique identifier, synonyms]

**Dressing**

exp Bandages/

exp Surgical Sponges/

exp Occlusive Dressings/

(bandage* or dressing* or gauze* or surgical sponge* or jelonet*).mp. [mp=title, abstract, original title, name of substance word, subject heading word, floating sub-heading word, keyword heading word, organism supplementary concept word, protocol supplementary concept word, rare disease supplementary concept word, unique identifier, synonyms]

**Osteosynthesis**

exp Bone Wires/

exp Internal Fixators/

exp Fracture Fixation, Internal/ or exp Fracture Fixation/ or exp Bone Nails/

exp Bone Nails/ or exp Fracture Fixation, Intramedullary/ or exp Fracture Fixation, Internal/

exp Fracture Fixation, Internal/ or exp Bone Plates/ or exp Bone Screws/

(osteosynthes* or fracture fixat* or skelet* fixat* or fracture* reduction* or intramedullar* nail* or internal fixat* or bone* plat* or bone* screw* or bone* nail* or bone* pin* or fracture* plat* or fracture* screw* or fracture* nail* or fracture* pin* or skelet* plat* or skelet* screw* or skelet* nail* or skelet* pin* or bone* wire* or k*wire* or Kirschner wire* or cerclage or tension band* or rod* or stabil*).mp. [mp=title, abstract, original title, name of substance word, subject heading word, floating sub- heading word, keyword heading word, organism supplementary concept word, protocol supplementary concept word, rare disease supplementary concept word, unique identifier, synonyms]

**Infection**

Coinfection/

exp Abscess/
exp Suppuration/
exp Bone Diseases/
exp Osteomyelitis/
exp Equipment Contamination/

exp Sepsis/

exp Surgical Wound Infection/ or exp Cross Infection/ or exp Wound Infection/ or exp Infection/

(infection* or infect* bone* or fracture* infect* or sepsis or blood poisoning* or soft tissue* infection* or suppuration* or pus* or abscess* or co*infection* or coinfect* or equipment* contamination* or osteomyelit*).mp. [mp=title, abstract, original title, name of substance word, subject heading word, floating sub-heading word, keyword heading word, organism supplementary concept word, protocol supplementary concept word, rare disease supplementary concept word, unique identifier, synonyms]

## EmBase

**NPWT**

exp suction catheter/ or exp suction canister/ or exp suction drainage/ or exp suction/ or exp suction drain/ or exp patient care suction apparatus/ or exp suction pump/

exp vacuum extraction/ or exp vacuum aspiration/ or exp vacuum extractor/ or exp vacuum pump/ or exp vacuum-formed retainer/ or exp vacuum assisted closure device/ or exp vacuum assisted closure/ or exp vacuum/ or exp vacuum therapy device/

(npwt or negative*pressure* wound therap* or negative*pressure* dressing* or topical* negative*pressure* therap* or vac or vacuum* or suction*).mp. [mp=title, abstract, heading word, drug trade name, original title, device manufacturer, drug manufacturer, device trade name, keyword, floating subheading word, candidate term word]

**Dressing**

exp gauze dressing/

exp jelonet/

exp surgical sponge/

exp elastic adhesive bandage/ or exp zinc oxide bandage/ or exp adhesive bandage/ or exp compression bandage/ or exp cotton bandage/ or exp bandage/ or exp support bandage/ or exp crepe bandage/

exp hydrocolloid dressing/ or exp pressure dressing/ or exp foam dressing/ or exp hydrogel dressing/ or exp occlusive dressing/ or exp silicone dressing/ or exp silver dressing/ or exp transparent dressing/ or exp antimicrobial dressing/ or exp gauze dressing/ or exp alginate dressing/ or exp silicone foam dressing/ or exp wound dressing/ or exp hemostatic dressing/

(bandage* or dressing* or gauze* or jelonet* or surgical sponge*).mp. [mp=title, abstract, heading word, drug trade name, original title, device manufacturer, drug manufacturer, device trade name, keyword, floating subheading word, candidate term word]

**Osteosynthesis**

exp osteosynthesis material/ or exp osteosynthesis/ or exp compression osteosynthesis/

exp fracture fixation/
exp bone plate/
exp plate fixation/
exp dynamic stabilization device/ exp bone screw/

exp intramedullary nailing/ or exp bone nail/ or exp intramedullary nail/

exp internal fixator/

exp bone pin/

exp Kirschner wire/ or exp bone wire/

exp cerclage applier/ or exp cerclage/ or exp orthopedic cerclage applier/

(osteosynthes* or fracture fixat* or skelet* fixat* or fracture* reduction* or intramedullar* nail* or internal fixat* or bone* plat* or bone* screw* or bone* nail* or bone* pin* or fracture* plat* or fracture* screw* or fracture* nail* or fracture* pin* or skelet* plat* or skelet* screw* or skelet* nail* or skelet* pin* or bone* wire* or k*wire* or Kirschner wire* or cerclage or tension band* or rod* or stabil*).mp. [mp=title, abstract, heading word, drug trade name, original title, device manufacturer, drug manufacturer, device trade name, keyword, floating subheading word, candidate term word]

**Infection**

exp infection/ or exp device infection/ or exp cross infection/

exp wound infection/

exp mixed infection/ or exp superinfection/ or exp secondary infection/ or exp bacterial infection/

exp bone infection/

exp soft tissue infection/

exp surgical infection/

exp postoperative complication/ or exp infection complication/

exp contamination/ or exp bacterium contamination/ or exp medical device contamination/ or exp microbial contamination/

exp medical device contamination/ or exp equipment/

exp osteomyelitis/ or exp chronic osteomyelitis/

exp bone disease/

exp pus/

exp abscess/ or exp skin abscess/ or exp abscess drainage/

exp sepsis/
exp suppuration/

(infection* or infect* bone* or fracture* infect* or sepsis or blood poisoning* or soft tissue* infection* or suppuration* or pus* or abscess* or co*infection* or coinfect* or equipment* contamination* or osteomyelit*).mp. [mp=title, abstract, heading word, drug trade name, original title, device manufacturer, drug manufacturer, device trade name, keyword, floating subheading word, candidate term word]

## Scopus

**NPWT**

( TITLE-ABS-KEY ( ( npwt )  OR  ( "negative*pressure* wound therap*" )  OR  ( "negative*pressure* dressing*" )  OR  ( "topical* negative*pressure* therap*" )  OR  ( vac )  OR  ( vacuum* )  OR  ( suction* ) ) )

**Dressing**

( TITLE-ABS-KEY ( ( bandage* ) OR ( dressing* ) OR ( gauze* ) OR ( jelonet* ) OR ( surgical AND sponge* ) )

**Osteosynthesis**

( TITLE-ABS-KEY ( ( osteosynthes* )  OR  ( "fracture fixat*" )  OR  ( "skelet* fixat*" )  OR  ( "fracture* reduction*" )  OR  ( "intramedullar* nail*" )  OR  ( "internal fixat*" )  OR  ( "bone* plat*" )  OR  ( "bone* screw*" )  OR  ( "bone* nail*" )  OR  ( "bone* pin*" )  OR  ( "fracture* plat*" )  OR  ( "fracture* screw*" )  OR  ( "fracture* nail*" )  OR  ( "fracture* pin*" )  OR  ( "skelet* plat*" )  OR  ( "skelet* screw*" )  OR  ( "skelet* nail*" )  OR  ( "skelet* pin*" )  OR  ( "bone* wire*" )  OR  ( "k*wire*" )  OR  ( "Kirschner wire*" )  OR  ( cerclage )  OR  ( "tension band*" )  OR  ( rod* )  OR  ( stabil* ) ) )

**Infection**

( TITLE-ABS-KEY ( ( infection* )  OR  ( "infect* bone*" )  OR  ( "fracture* infect*" )  OR  ( sepsis )  OR  ( "blood poisoning*" )  OR  ( "soft tissue* infection*" )  OR  ( suppuration* )  OR  ( pus* )  OR  ( abscess* )  OR  ( "co*infection*" )  OR  ( coinfect* )  OR  ( "equipment* contamination*" )  OR  ( osteomyelit* ) ) )

# Appendix B – Full search in Scopus

Search combination for Scopus on “NPWT AND osteosyntheses AND infection” was as follows:

( TITLE-ABS-KEY ( ( npwt ) OR ( "negative*pressure* wound therap*" ) OR ( "negative*pressure* dressing*" ) OR ( "topical* negative*pressure* therap*" ) OR ( vac ) OR ( vacuum* ) OR ( suction* ) ) ) AND ( TITLE-ABS-KEY ( ( osteosynthes* ) OR ( "fracture fixat*" ) OR ( "skelet* fixat*" ) OR ( "fracture* reduction*" ) OR ( "intramedullar* nail*" ) OR ( "internal fixat*" ) OR ( "bone* plat*" ) OR ( "bone* screw*" ) OR ( "bone* nail*" ) OR ( "bone* pin*" ) OR ( "fracture* plat*" ) OR ( "fracture* screw*" ) OR ( "fracture* nail*" ) OR ( "fracture* pin*" ) OR ( "skelet* plat*" ) OR ( "skelet* screw*" ) OR ( "skelet* nail*" ) OR ( "skelet* pin*" ) OR ( "bone* wire*" ) OR ( "k*wire*" ) OR ( "Kirschner wire*" ) OR ( cerclage ) OR ( "tension band*" ) OR ( rod* ) OR ( stabil* ) ) ) AND ( TITLE-ABS-KEY ( ( infection* ) OR ( "infect* bone*" ) OR ( "fracture* infect*" ) OR ( sepsis ) OR ( "blood poisoning*" ) OR ( "soft tissue* infection*" ) OR ( suppuration* ) OR ( pus* ) OR ( abscess* ) OR ( "co*infection*" ) OR ( coinfect* ) OR ( "equipment* contamination*" ) OR ( osteomyelit* ) ) )

No limitations were used.

Search combination for Scopus on “dressing AND osteosyntheses AND infection” was as follows:

( TITLE-ABS-KEY ( ( bandage* ) OR ( dressing* ) OR ( gauze* ) OR ( jelonet* ) OR ( surgical AND sponge* ) ) AND PUBYEAR < 2021 ) AND ( TITLE-ABS-KEY ( ( osteosynthes* ) OR ( fracture AND fixat* ) OR ( skelet* AND fixat* ) OR ( fracture* AND reduction* ) OR ( intramedullar* AND nail* ) OR ( internal AND fixat* ) OR ( bone* AND plat* ) OR ( bone* AND screw* ) OR ( bone* AND nail* ) OR ( bone* AND pin* ) OR ( fracture* AND plat* ) OR ( fracture* AND screw* ) OR ( fracture* AND nail* ) OR ( fracture* AND pin* ) OR ( skelet* AND plat* ) OR ( skelet* AND screw* ) OR ( skelet* AND nail* ) OR ( skelet* AND pin* ) OR ( bone* AND wire* ) OR ( k*wire* ) OR ( kirschner AND wire* ) OR ( cerclage ) OR ( tension AND band* ) OR ( rod* ) ) AND PUBYEAR < 2021 ) AND ( TITLE-ABS-KEY ( ( infection* ) OR ( infect* AND bone* ) OR ( fracture* AND infect* ) OR ( sepsis ) OR ( blood AND poisoning* ) OR ( soft AND tissue* AND infection* ) OR ( suppuration* ) OR ( pus* ) OR ( abscess* ) OR ( co*infection* ) OR ( coinfect* ) OR ( equipment* AND contamination* ) OR ( osteomyelit* ) ) AND PUBYEAR < 2021 )

Search limitations were publications until 2021.

# Appendix C – Question description - Table 6 – Risk of bias case reports

**Case Reports Critical Appraisal Tool**

Answers: Yes, No, Unclear or Not/Applicable

**1. Were patient’s demographic characteristics clearly described?**

Does the case report clearly describe patient's age, sex, race, medical history, diagnosis, prognosis, previous treatments, past and current diagnostic test results, and medications? The setting and context may also be described.

**2. Was the patient’s history clearly described and presented as a timeline?**

A good case report will clearly describe the history of the patient, their medical, family and psychosocial history including relevant genetic information, as well as relevant past interventions and their outcomes. (CARE Checklist 2013)

**3. Was the current clinical condition of the patient on presentation clearly described?**

The current clinical condition of the patient should be described in detail including the uniqueness of the condition/disease, symptoms, frequency and severity. The case report should also be able to present whether differential diagnoses was considered.

**4. Were diagnostic tests or methods and the results clearly described?**

A reader of the case report should be provided sufficient information to understand how the patient was assessed. It is important that all appropriate tests are ordered to confirm a diagnosis and therefore the case report should provide a clear description of various diagnostic tests used (whether a gold standard or alternative diagnostic tests). Photographs or illustrations of diagnostic procedures, radiographs, or treatment procedures are usually presented when appropriate to convey a clear message to readers.

**5. Was the intervention(s) or treatment procedure(s) clearly described?**

It is important to clearly describe treatment or intervention procedures as other clinicians will be reading the paper and therefore may enable clear understanding of the treatment protocol. The report should describe the treatment/intervention protocol in detail; for e.g. in pharmacological management of dental anxiety - the type of drug, route of administration, drug dosage and frequency, and any side effects.

**6. Was the post-intervention clinical condition clearly described?**

A good case report should clearly describe the clinical condition post-intervention in terms of the presence or lack thereof symptoms. The outcomes of management/treatment when presented as images or figures would help in conveying the information to the reader/clinician.

**7. Were adverse events (harms) or unanticipated events identified and described?**

With any treatment/intervention/drug, there are bound to be some adverse events and in some cases, they may be severe. It is important that adverse events are clearly documented and described, particularly when a new or unique condition is being treated or when a new drug or treatment is used. In addition, unanticipated events, if any that may yield new or useful information should be identified and clearly described.

**8. Does the case report provide takeaway lessons?**

Case reports should summarize key lessons learned from a case in terms of the background of the condition/disease and clinical practice guidance for clinicians when presented with similar cases.

# Appendix D – Question description - Table 7 – Risk of bias case series

**Critical Appraisal Checklist for Case Series**

Answers: Yes, No, Unclear or Not/Applicable

**1. Were there clear criteria for inclusion in the case series?**

The authors should provide clear inclusion (and exclusion criteria where appropriate) for the study participants. The inclusion/exclusion criteria should be specified (e.g., risk, stage of disease progression) with sufficient detail and all the necessary information critical to the study.

**2. Was the condition measured in a standard, reliable way for all participants included in the case series?**

The study should clearly describe the method of measurement of the condition. This should be done in a standard (i.e. same way for all patients) and reliable (i.e. repeatable and reproducible results) way.

**3. Were valid methods used for identification of the condition for all participants included in the case series?**

Many health problems are not easily diagnosed or defined and some measures may not be capable of including or excluding appropriate levels or stages of the health problem. If the outcomes were assessed based on existing definitions or diagnostic criteria, then the answer to this question is likely to be yes. If the outcomes were assessed using observer reported, or self-reported scales, the risk of over- or under-reporting is increased, and objectivity is compromised. Importantly, determine if the measurement tools used were validated instruments as this has a significant impact on outcome assessment validity.

**4. Did the case series have consecutive inclusion of participants?**

Studies that indicate a consecutive inclusion are more reliable than those that do not. For example, a case series that states ‘we included all patients (24) with osteosarcoma who presented to our clinic between March 2005 and June 2006’ is more reliable than a study that simply states ‘we report a case series of 24 people with osteosarcoma.’

**5. Did the case series have complete inclusion of participants?**

The completeness of a case series contributes to its reliability (1). Studies that indicate a complete inclusion are more reliable than those that do not. A stated above, a case series that states ‘we included all patients (24) with osteosarcoma who presented to our clinic between March 2005 and June 2006’ is more reliable than a study that simply states ‘we report a case series of 24 people with osteosarcoma.’

**6. Was there clear reporting of the demographics of the participants in the study?**

The case series should clearly describe relevant participant’s demographics such as the following information where relevant: participant’s age, sex, education, geographic region, ethnicity, time period, education.

**7. Was there clear reporting of clinical information of the participants?**

There should be clear reporting of clinical information of the participants such as the following information where relevant: disease status, comorbidities, stage of disease, previous interventions/treatment, results of diagnostic tests, etc.

**8. Were the outcomes or follow-up results of cases clearly reported?**

The results of any intervention or treatment should be clearly reported in the case series. A good case study should clearly describe the clinical condition post-intervention in terms of the presence or lack of symptoms. The outcomes of management/treatment when presented as images or figures can help in conveying the information to the reader/clinician. It is important that adverse events are clearly documented and described, particularly a new or unique condition is being treated or when a new drug or treatment is used. In addition, unanticipated events, if any that may yield new or useful information should be identified and clearly described.

**9. Was there clear reporting of the presenting site(s)/clinic(s) demographic information?**

Certain diseases or conditions vary in prevalence across different geographic regions and populations (e.g. women vs. men, sociodemographic variables between countries). The study sample should be described in sufficient detail so that other researchers can determine if it is comparable to the population of interest to them.

**10. Was statistical analysis appropriate?**

As with any consideration of statistical analysis, consideration should be given to whether there was a more appropriate alternate statistical method that could have been used. The methods section of studies should be detailed enough for reviewers to identify which analytical techniques were used and whether these were suitable.
